# Integrated analysis of somatic mutations and immune microenvironment of multiple regions in breast cancers

**DOI:** 10.18632/oncotarget.18790

**Published:** 2017-06-28

**Authors:** Taigo Kato, Jae-Hyun Park, Kazuma Kiyotani, Yuji Ikeda, Yasuo Miyoshi, Yusuke Nakamura

**Affiliations:** ^1^ Department of Medicine, The University of Chicago, Chicago, IL 60637, USA; ^2^ Divison of Breast and Endocrine, Department of Surgery, Hyogo College of Medicine, Hyogo 663-8501, Japan; ^3^ Department of Surgery, The University of Chicago, Chicago, IL 60637, USA

**Keywords:** breast cancer, heterogeneity, T cell receptor, non-synonymous mutation, neoantigen

## Abstract

Next-generation sequencing technology enables us to analyze the complexity of intra- and inter-tumoral heterogeneity, which may influence to prognosis of cancer patients. In this study, we collected surgically-resected tumor tissues from five breast cancer patients and characterized three different portions of individual tumors through somatic mutation analysis by whole exome sequencing, T cell receptor beta (TCRB) repertoire analysis of tumor-infiltrating lymphocytes (TILs), and the expression analysis of immune-related genes at 15 different sites. This integrated analysis revealed distinguished patterns of somatic mutations and TIL clonotypes in the three portions of each tumor, implying that the tumor heterogeneity is comprised by spatially different somatic mutations as well as the presence of diverse T cell clones. Furthermore, higher numbers of the non-synonymous somatic mutations were significantly correlated with the higher ratio of *GZMA/TCRB* expression (*P* = 0.0004), implying that high somatic mutation load in tumor might be correlated to the number of immunogenic antigens and then functionally activate TILs with higher cytolytic activity. Our findings suggest that breast cancers comprise with very complex tumor heterogeneity by the spatially different mutational landscape and immune microenvironment, and that mutation/neoantigen load may be strongly correlated with induction of cancer-specific TILs and affect the immune microenvironment in breast tumors.

## INTRODUCTION

Breast cancer is the most common female cancer and the second leading cause of cancer death among women in the United States. Although the 5-year disease-specific survival has been improved from 74.6 % in 1975 - 1979 to 90.6 % in 2006 [1] due to development of an early-detection screening systems as well as systemic treatments, some breast cancer cases are still detected at an advanced stage and show a higher mortality rate due to drug resistance and a high rate of recurrence [2].

The intra-tumoral heterogeneity in cancers is often a subject to discussion when molecular targeted therapies are not effective for all selected patients [3, 4]. Recently, the whole exome sequencing approach from multiple sites of individual tumors has revealed remarkable intra-tumoral heterogeneity in terms of the mutational landscape in several types of cancer, which harbors evolutionally accumulated somatic mutations in individual tumor regions [5, 6]. Therefore, in-depth characterization and understanding of such molecular diversity in different tumor regions may improve diagnosis and design of effective treatment strategies.

Tumor-infiltrating lymphocytes (TILs) are known to be one of the important prognostic factors in various types of human cancer [7, 8]. Currently, immunohistochemical analysis including CD3 and CD8 staining is widely used to evaluate infiltration of T cells into tumors, and flow cytometry analysis enables us to perform the quantification of T cell subpopulations [9]. However, these approaches are still far from detailed characterization of extremely diverse T cell repertoires which may recognize a wide variety of cancer-specific antigens. Therefore, aiming to the comprehensive analysis of T cell clonality and activities in tumors, we established and applied our T cell receptor (TCR) repertoire analysis method with a next-generation sequencer using RNAs extracted from blood, ascites and tumors [10–14].

In the present study, we unraveled intra- and inter-tumoral heterogeneity in multiregional samples from 5 breast cancer patients through TCR sequencing, whole exome analysis as well as expression analysis of immune-related genes. Here, we report that TILs in individual regions had unique characteristics along with somatic mutation patterns. We also found the *HLA-A* expression level in tumors may have a correlation to higher cytolytic activity of TILs as well as the composition of TCR repertoire in tumors. Given these findings, deciphering the tumor heterogeneity in both genetics and immune aspects may have important implications for future biomarker discovery and cancer treatments by identification of neoantigens and their corresponding T cell clones.

## RESULTS

### Intra-tumoral genetic heterogeneity in three different portions of breast cancer

**Figure 1:**
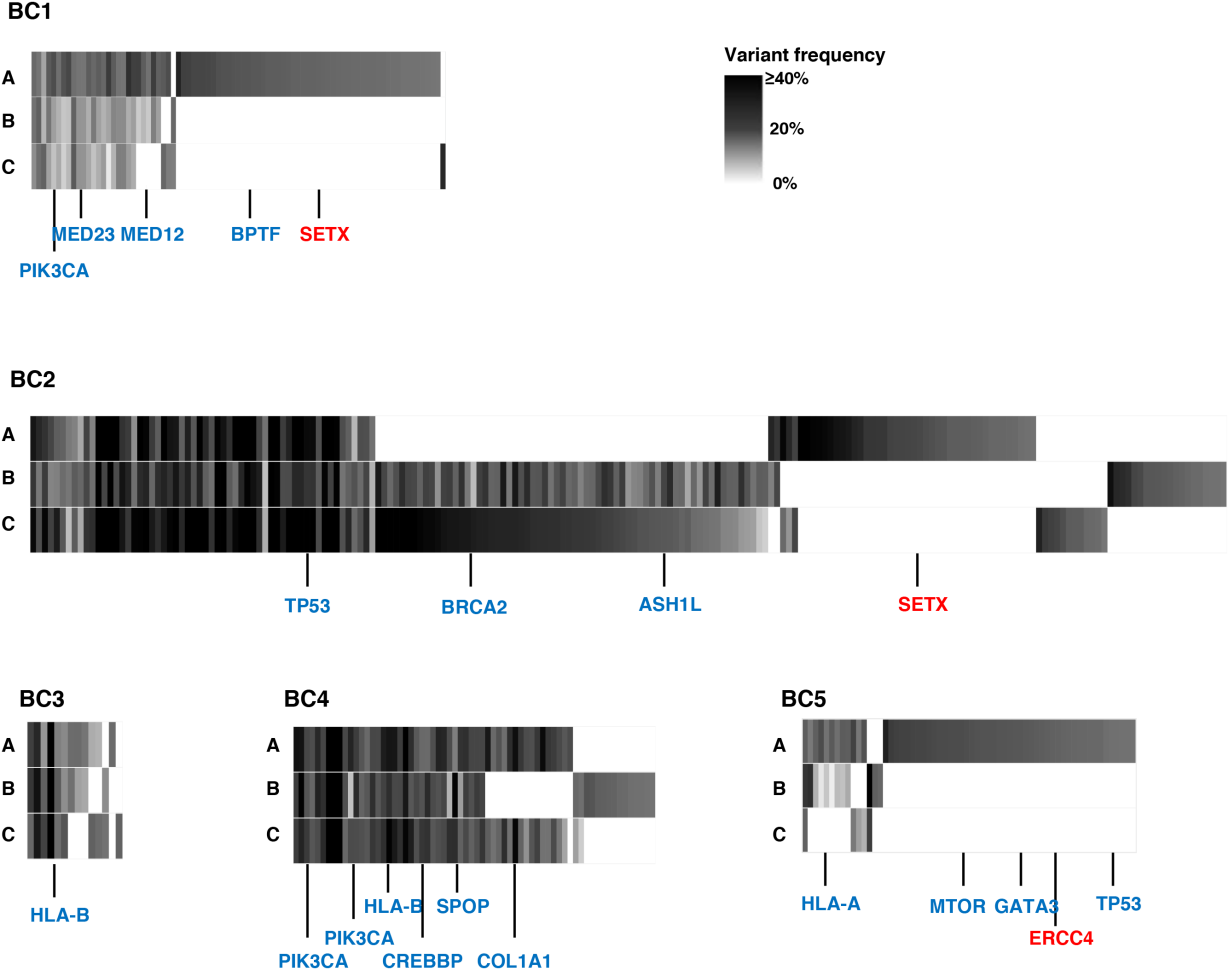
Genetic intra-tumoral heterogeneity in five breast tumors Multiregional profiles of mutations were visualized as heat maps (blue colored genes: driver gene mutations in many types of cancers including breast cancer; red colored genes: DNA mismatch repair genes). *ASH1L*: ASH1 Like Histone Lysine Methyltransferase; *BPTF*: Bromodomain PHD Finger Transcription Factor; *BRCA2*: Breast Cancer Susceptibility gene II; *COL1A1*: Collagen Type I Alpha I chain; *CREBBP*: CREB Binding Protein; ERCC4: ERCC excision repair 4; *GATA3*: GATA Binding Protein 3; *HLA-A*: Human Leukocyte Antigen A; *HLA-B*: Human Leukocyte Antigen B; *MED12*: Mediator Complex Subunit 12; *MED23*: Mediator Complex Subunit 23; *MTOR*: Mechanistic target of rapamycin; *PIK3CA*: Phosphatidylinositol-4,5-Bisphosphate 3-Kinase Catalytic Subunit Alpha; *SETX*: Senataxin; *SPOP*: Speckle Type BTB/POZ Protein; *TP53*: Tumor Protein 53.

**Figure 2:**
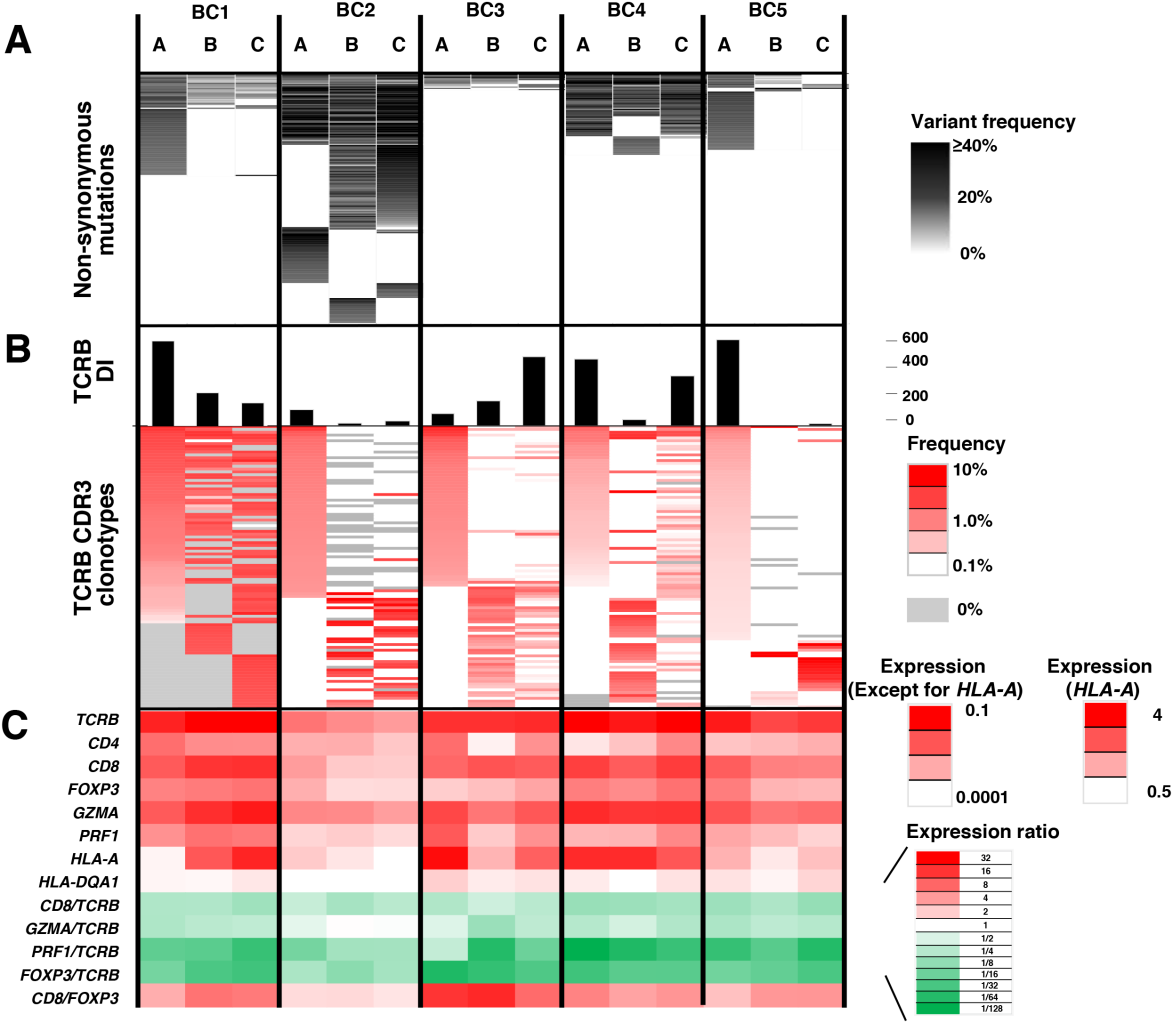
Integrated analysis of non-synonymous mutations, TCRB repertoire and immune-related gene expression levels for intra-tumoral heterogeneity Integrated data from three different portions **(A, B, C)** of the five breast tumors. **(A)** Commonality of non-synonymous mutations. **(B)** TCRB diversity index (DI) and heatmaps of TCRB CDR3 clonotypes which were sorted according to their frequencies (higher to lower) in the order of tumor portions, **(A, B** and **C)**. **(C)** the transcriptional levels of immune-related genes. The expression level of each gene was calculated relative to that of *GAPDH*.

To examine intra- and inter-tumoral genetic heterogeneity in breast cancer tissues, we performed the whole-exome sequencing using genomic DNAs extracted from three separated portions (A, B, C) of surgically-resected tumors. The clinical characteristics of all patients are summarized in Table 1. We obtained an average sequencing depth of 82.3× per base, and identified a total of 498 non-silent mutations and insertions/deletions (indels) (15-252 mutations per sample, Supplementary Table 1). We found that 1.6% - 52.9% of somatic mutations, including well-known cancer driver genes such as *PIK3CA* and *TP53* that have been reported to be generally common in parental clones in many types of cancer, were shared among three portions (Figure 1 and Supplementary Figure 1) [15–18]. In contrast, some portions of cancer tissues such as BC1-A, BC2-A and BC5-A had their unique mutations including DNA mismatch repaired genes, *SETX* and *ERCC4* (Figure 1), which might be acquired during the clonal evolution for cancer cells and contributed to high genetic intra-tumoral heterogeneity in these tumor portions. We subsequently selected only non-synonymous mutations (Figure 2A) to examine correlation between the genetic heterogeneity and immune signature in each tumor sample. With respect to predicted potential neoantigen epitopes, which were generated by non-synonymous somatic mutations, we identified 0 to 51 potential neoantigen candidates (the binding affinity to either of HLA-A, B and C molecules of less than 500 nM, an average number of 22.9) in each tumor portion (Supplementary Figure 2). We detected unique neoantigens in each portion of individual tumor, whereas in two of five cases (BC2 and BC4), we found neoantigens which were shared by all three portions.

**Table 1:** Clinical information of 5 breast cancer patients

Patients	Age	Subtype	Tumor size (mm^2^)	Menopause	Birth history	Neo-hormone therapy	Procedure	Pathology	Stage	Recurrence	Status	Follow-up duration (dy)
**BC1**	**72**	**TNBC**	**29 × 15**	**post**	**3**	**-**	**LtBt**	**Lobular carcinoma**	**2B**	**+** **(bone metastasis)**	**Alive**	**614**
**BC2**	**94**	**TNBC**	**19 × 10**	**post**	**4**	**-**	**RtBp**	**Scirrhous carcinoma**	**2A**	**-**	**Alive**	**359**
**BC3**	**41**	**Luminal A**	**20 × 15**	**pre**	**3**	**-**	**LtBt**	**Scirrhous carcinoma**	**2A**	**-**	**Alive**	**685**
**BC4**	**47**	**Luminal A**	**19 × 10**	**post**	**0**	**-**	**LtBt**	**Scirrhous carcinoma**	**2A**	**-**	**Alive**	**703**
**BC5**	**75**	**Luminal A**	**15 × 8**	**post**	**2**	**Aromatase** **Inhibitor**	**LtBt**	**Scirrhous carcinoma**	**2A**	**-**	**Alive**	**720**

TNBC: triple-negative breast cancer, Luminal A: luminal A type of breast cancer, Lt: left, Rt: right, Bt: total mastectomy, Bp: partial mastectomy.

### Heterogeneity of TCR repertoire and immune-related gene signature in three different portions of breast cancer

To further characterize T cell repertoire and their relationship with the intra-tumoral heterogeneity of breast cancer, we performed TCRB repertoire analysis using the next generation sequencing method and estimated the frequency of individual TCRB CDR3 clonotypes. Through cDNA sequencing of TCRB, we obtained total sequence reads of 2,481,978 ± 1,744,671 (average ± one standard deviation) mapped to V, D, J, and C segments for TCRB. From these TCRB reads, we identified 80,604 ± 87,011 unique CDR3 clonotypes for TCRB (Supplementary Table 2) and calculated the TCRB diversity index in each portion of tumors (Figure 2B). After sorting out the most abundant 100 CDR3 clonotypes according to their frequencies in tumor tissue samples (Figure 2B), we found common CDR3 clonotypes (detected in all three portions) as well as spatially unique CDR3 clonotypes (detected in only one portion), indicating the intra-tumoral heterogeneity in the immune signature. Similarly, gene expression analysis of immune-related genes showed distinguished expression patterns of multiple immune-related genes, such as *TCRB*, *CD4*, *CD8*, *FOXP3*, granzyme A (*GZMA*), perforin 1 (*PRF1*), *HLA-A* and *HLA-DQA1*, and the ratios of *CD8/TCRB*, *GZMA/TCRB*, *PRF1/TCRB*, *FOXP3/TCRB* and *CD8/FOXP3* among three different portions in individual tumors (Figure 2C), further suggesting that the immune microenvironment is spatially heterogeneous in these five breast cancer cases.

### Clustering analysis to assess intra-tumoral heterogeneity between somatic mutations and TCRB repertoires in breast cancer

**Figure 3:**
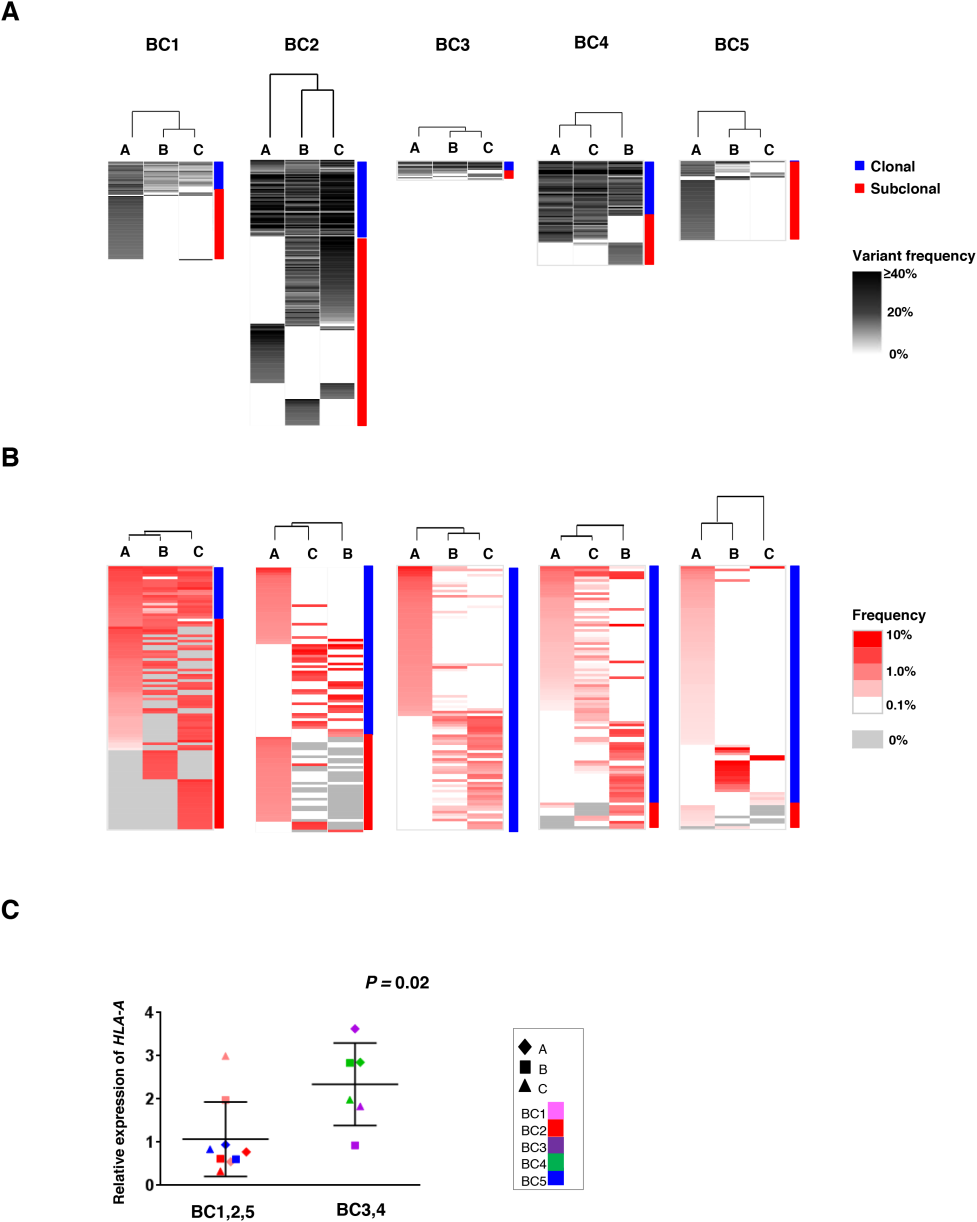
Clustering analysis of the multiregional non-synonymous mutation profiles and TCRB repertoires Three different portions of individual tumors were shown as hierarchical clustering by calculating their similarity in the datasets of non-synonymous mutations **(A)** and TCRB repertoires **(B)**. Vertical length of dendrogam indicates the similarity between two datasets. **(C)** Comparison of *HLA-A* expression levels in the three tumor tissues where clustering patterns based on somatic mutations were different from those based on TCRB repertoire (*left*) and those in tumors showing the similar clustering patterns (*right*).

To address the correlation between the intra-tumoral heterogeneity in somatic mutation patterns and that in TCRB repertoires among the three tumor portions, we conducted unsupervised clustering analysis by calculating the similarity index (SI) of somatic mutation profiles as well as TCRB profiles in the three portions. As shown in Figure 3A, while common somatic mutations in all three portions (clonal mutations) were detected, some mutations were uniquely observed in one or two tumor portions (subclonal mutations). Proportions of the subclonal mutations varied among the patients as 64.3 ± 21.2 %. Interestingly, 61 of 62 mutations were subclonal mutations in the BC5 case, indicating the very high level of the intra-tumoral heterogeneity probably due to clonal selection of resistant cancer cell subpopulations through pre-treatment of aromatase inhibitor. The clustering patterns based on TCRB repertoires of the three different portions (Figure 3B) were quite similar to those based on somatic mutations in two cases (BC3 and BC4) where common somatic mutations were more frequently detected. In contrast, other three cases (BC1, BC2 and BC5) showed different clustering patterns between somatic mutations and TCRB clonotypes. To clarify this inconsistency, we examined the *HLA-A* expression level as HLA class I molecules are required for the presentation of tumor-associated antigens to cytotoxic CD8+ T cells. Interestingly, we found the transcriptional level of *HLA-A* was significantly lower in the three cases that showed the inconsistency between the clustering patterns of somatic mutations and those of TCRB repertoires (*P* = 0.02, Figure 3C). The *HLA-A* expression level also showed a positive correlation with *CD8*, *GZMA* and *PRF1* expression levels in tumors (Supplementary Figure 3). Therefore, these results indicated that *HLA-A* expression could be one of important determinants to define immune microenvironment.

### Correlation between non-synonymous somatic mutations and immune-related gene expressions

**Figure 4:**
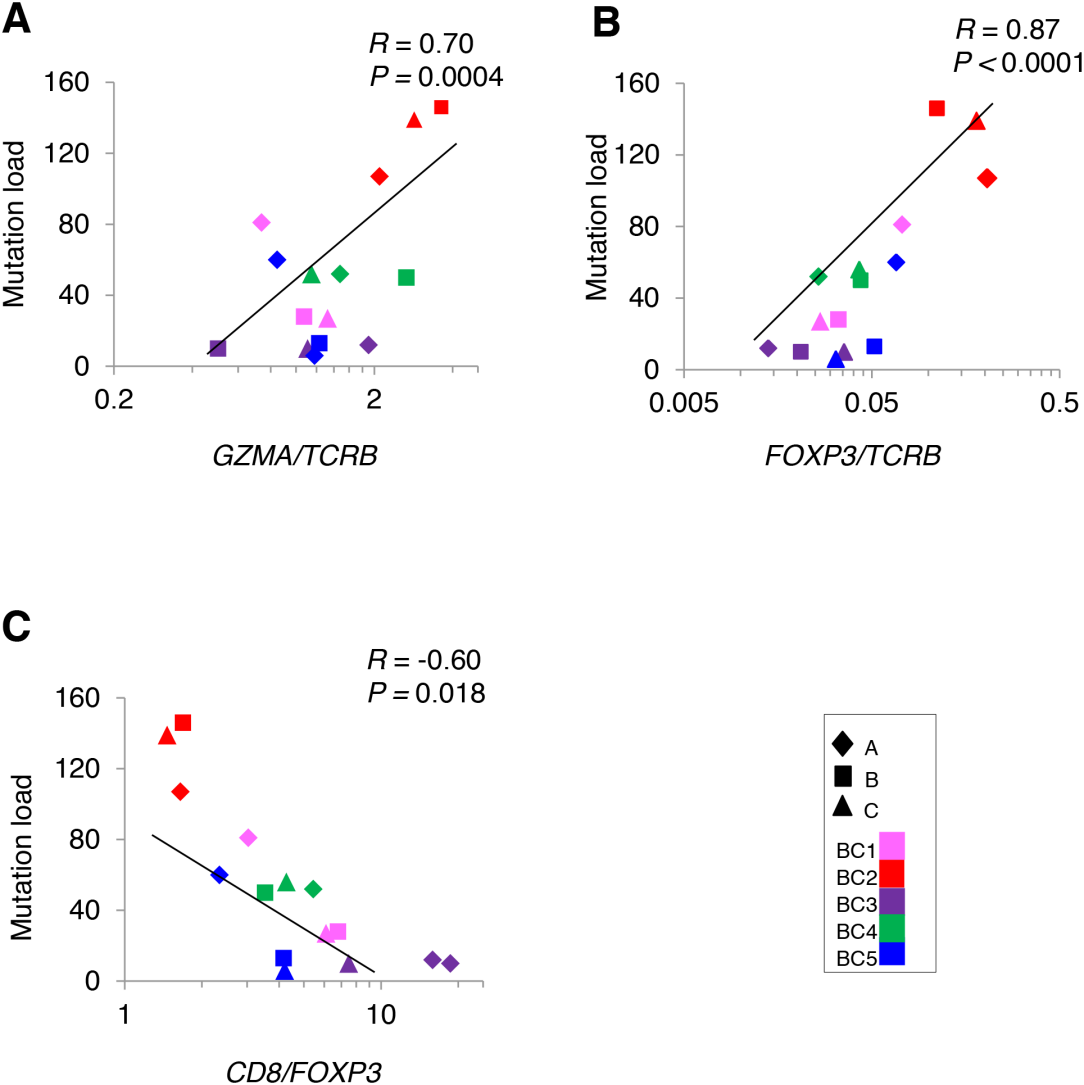
Correlation analysis between non-synonymous mutations and the mRNA expression level of immune-related genes in tumors Correlation of non-synonymous mutation loads in five breast tumors (*N* = 15) to the ratios of **(A)** *GZMA/TCRB* **(B)** *FOXP3/TCRB* **(C)** *CD8/FOXP3*.

To analyze a relationship between non-synonymous somatic mutations and immune microenvironment, we compared the number of non-synonymous mutations with mRNA expression levels of immune-related genes (Figure 4). The non-synonymous mutation load showed a strong positive correlation with *GZMA/TCRB* ratio (Figure 4A), indicating that higher numbers of somatic mutation were correlated with higher cytolytic activity of T cells, likely to be CD8+ cells. We also found the higher numbers of non-synonymous mutations were correlated with higher ratio of an immune suppressive marker *FOXP3/TCRB* along with lower *CD8/FOXP3* ratio (Figure 4B, 4C), which might reflect counter-immunosuppressive mechanisms to protect cancer cells from a host immure attack. These results indicated a possibility that accumulation of non-synonymous mutations probably caused higher cytolytic activity of infiltrated TILs along with immune escape mechanism by cancer cells.

## DISCUSSION

Drug resistance is one of the most important issues in the clinical management of cancer patients at an advanced stage or with recurrent metastatic tumors. Genetic heterogeneity within or between tumors as well as their tumor microenvironment are considered to play critical roles in the drug resistance. By means of next generation sequencing technologies, we are able to examine intra- and inter-tumoral heterogeneity in depth [5, 6]. However, such heterogeneity has been focused on somatic mutational landscapes, but not much on the immune environment. Hence, in this study, we characterized the T cell repertoires of tumor-infiltrating lymphocytes and expression levels of immune-related genes, and compared them with somatic mutation profiles of three separated portions of individual tumor tissues and found some evidences that may evoke further understanding of various aspects of the heterogeneity in breast tumors.

Firstly, TILs revealed a relatively large diversity among the different portions in a single tumor according to somatic mutation profiles. In addition, the clustering analysis showed the similarities between TCRB clonal patterns and non-synonymous mutational patterns in two cases (BC3 and BC4), but in the remaining three cases, BC1, BC2 and BC5, the clustering patterns based on non-synonymous mutation among three portions were different from those based on TCRB clonotypes. Interestingly, we found that *HLA-A* expression levels in these three cancer tissues were very low. Hence, we considered poor immune responses (particularly CD8 responses) in these cancer tissues, and then the clustering patterns based on T cell clonotypes lost the correlation with those based on non-synonymous mutations. We previously reported that intra-tumoral high expression of *HLA-A* might be one of the predictive markers for clinical responses to anti PD-1 therapy in metastatic melanoma [19]. Given that CD8+ T cells play a central role in cancer immunosurveillance and the loss or down-regulation of HLA class I molecule expression is one of major immune escape mechanisms of cancer cells [20, 21], our findings collectively indicate that HLA class I expression levels should be considered as one of important determinants for the intra-tumoral heterogeneity of TILs.

Secondly, likely as The Cancer Genome Atlas (TCGA) data which showed the correlation between the increasing somatic mutation burden and T cell effector function [22], we confirmed that higher non-synonymous mutation load was significantly correlated with higher cytolytic activity of T cells as shown by increasing *GZMA/TCRB* expression ratio. We also found higher *FOXP3/TCRB* ratio in tumor portions was significantly correlated with higher mutation load, which indicates a kind of immune balance between the cytolytic T cells and regulatory T cells in these tumors where immune checkpoint blockades may effectively work by activating pre-existing anti-cancer immunity.

In summary, our integrated analysis from three different portions in individual tumors demonstrated that not only somatic mutational profiles, but also T cell clonotypes and expression levels of immune-related genes, which define immune microenvironment, revealed the relatively high levels of heterogeneity. Our data clearly indicate that the accumulation of integrated analysis of somatic mutation profiles and characterization of immune microenvironment is critically important in the further understanding of human cancers.

## MATERIALS AND METHODS

### Study design

Between January 2015 and March 2015, a total of five breast cancer patients were enrolled in this study and received surgery. Four cases had received no treatment before surgery and one had received letrozole and exemestane (aromatase inhibitors) for 3 months and 1.5 months respectively. The clinical characteristics of all patients are summarized in Table 1.

The study protocol was approved by the Institutional Review Board of University of Chicago (approval number 13-0797 and 13-0526) and Hyogo College of Medicine (approval number 106). All patients provided written informed consents.

### Whole-exome sequencing and data analysis

We selected three cancer cell-enriched portions from each of five frozen tumor tissues, and extracted genomic DNAs and total RNAs using AllPrep DNA/RNA mini kit (Qiagen, Valencia, CA). As germline control DNAs, genomic DNAs were extracted from peripheral blood mononuclear cells (PBMCs). Whole-exome libraries were prepared from 1,000 ng of genomic DNAs using SureSelectXT Human All Exon V5 kit (Agilent Technologies, Santa Clara, CA) and the prepared whole-exome libraries were sequenced by 100-bp paired-end reads on HiSeq2500 Sequencer (Illumina, San Diego, CA).

In the analysis of sequencing data, we firstly excluded low-quality reads (base quality of < 20 for more than 80% of bases) using FASTX toolkit (http://hannonlab.cshl.edu/fastx_toolkit/), and mapped sequence reads to the human reference genome GRCh37/hg19 using Burrows-Wheeler Aligner (BWA) (v0.7.10) [23]. Possible PCR duplicated reads were removed using Picard v1.91 (http://broadinstitute.github.io/picard/), and read pairs with a mapping quality of < 30 and with mismatches of more than 5% of nucleotides were also excluded. Finally, somatic variants (single nucleotide variations (SNVs) and indels) were called using the Fisher’s exact test-based method with the following parameters, (i) base quality of ≥ 15, (ii) sequence depth of ≥ 10, (iii) variant depth of ≥ 2, (iv) variant frequency in tumor of ≥ 10%, (v) variant frequency in normal of < 2%, and (vi) Fisher *P* value of < 0.05.[24] SNVs and indels were annotated based on RefGene using ANNOVAR as previously described [25, 26].

### Prediction of neoantigens

Based on whole-exome sequence data from the germline DNAs of breast cancer patients, HLA class I genotypes were estimated by OptiType algorithm [27]. Then, non-synonymous somatic mutations identified through the whole-exome sequencing data of 15 tumor samples were utilized for the prediction of the HLA genotypes-restricted neoantigens. Briefly, we examined all 8- to 11-mer peptides harboring each substituted amino acid by applying the filtering with the predicted binding affinity to HLA-A, B and C of <500 nM, using NetMHCv3.4 and NetMHCpanv2.8 software [11, 28–30].

### TCR sequencing and data analysis

Total RNAs from tumor tissues were isolated using AllPrep DNA/RNA Mini kit (Qiagen). Sequencing libraries of TCRB were prepared as described previously [11, 30] and subjected to sequencing on the Illumina Miseq platform, using 600 cycles Miseq Reagent Kit V3 (Illumina).

To identify V, D, J and C segments in individual TCRB sequencing reads, each of the sequence reads in FASTQ files were mapped to the reference sequences provided by IMGT/GENE-DB [31] using Bowtie2 aligner (Version 2.1.0) [32, 33]. To define amino acid sequences of complementarity determining region 3 (CDR3) in the TCRB, raw FASTQ files were also analyzed using Tcrip software [30].

### Gene expression analysis

cDNA was synthesized from tumor-derived RNAs using Superscript III first-strand synthesis kit (Invitrogen, Carlsbad, CA). The expression level of immune-related genes, *TCRB*, *CD4*, *CD8*, *FOXP3*, *GZMA*, *PRF1*, *HLA-A* and *HLA-DQA1*, were measured by real-time RT-PCR using Taqman gene expression assay (Life Technologies, Grand Island, NY) in the ABI ViiA 7 system (Applied Biosystems, Foster City, CA), according to the manufacturer’s instructions.

### Clustering analysis

To examine similarity (or distance) of datasets from the 3 different breast tumor regions, we conducted unsupervised hierarchical clustering analysis using Cluster 3.0 and TreeView software [34]. Briefly, the similarity metric was computed by the Pearson correlation coefficient of each somatic mutation or TCRB clonotypes, which then generated similarity index (SI) between two datasets based on the mean of all pairwise distances between two items (average linkage method). According to the SI and clustered nodes, dendrogram figures were generated by TreeView software.

### Statistical analysis

The diversity index (inverse Simpson’s index) in CDR3 sequences was calculated as follows:

$$1/D_{S}={\left[\frac{\sum_{i=1}^{K}n_{i}\left(n_{i}-1\right)}{N\left(N-1\right)}\right]}^{-1}$$

Where *K* is the total number of CDR3 clonotypes, *n_i_* is the number of sequences belonging to the *i*-th clonotype, and *N* is the total number of identified CDR3 sequences.

Pearson correlation (R) was used to analyze the association between all parameters examined. Statistical analysis was carried out using GraphPad Prism version 6.0 (GraphPad software, La Jolla, CA). *P* value of < 0.05 was considered to be statistically significant.

## SUPPLEMENTARY MATERIALS FIGURES AND TABLES





## Abbreviations

CDR3: complementary determining region 3
SI: similarity index
TCR: T cell receptor
TILs: tumor-infiltrating lymphocytes
