# Supplementary material for: Integrated analysis of somatic mutations and immune microenvironment of multiple regions in breast cancers
Source: Oncotarget. 2017 Jun 28;8(37):62029–38. doi: 10.18632/oncotarget.18790 (PMC5617483; doi:10.18632/oncotarget.18790)
Supplement: Supplementary file 1 [file oncotarget-08-62029-s001.pdf]

Integrated analysis of somatic mutations and immune microenvironment of multiple regions in breast cancers

SUPPLEMENTARY FIGURES AND TABLES

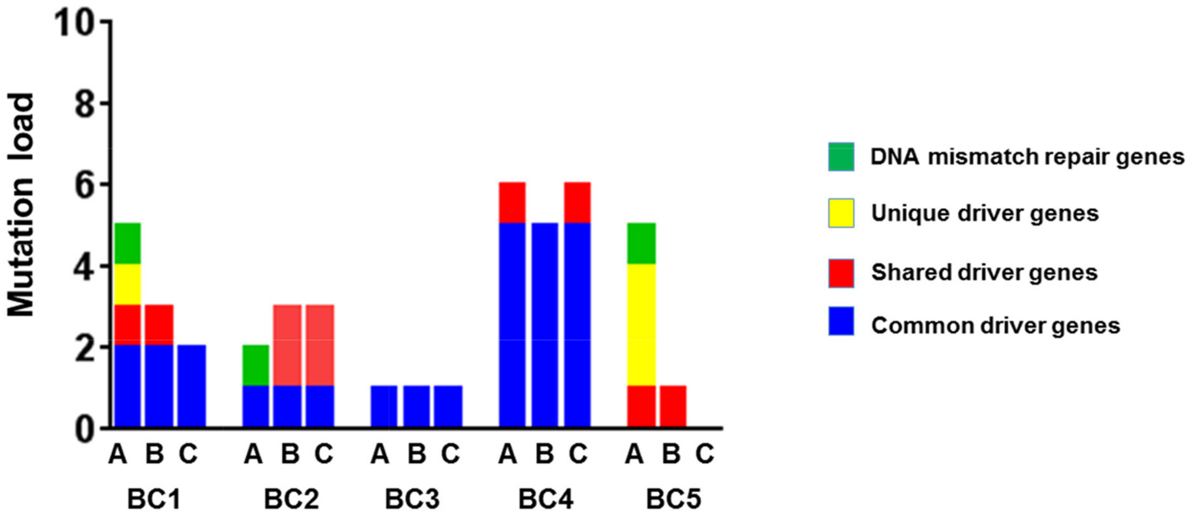

Supplementary Figure 1: The number of mutations having common driver genes in three portions (blue), shared driver genes in two portions (red), unique driver genes (yellow), and DNA mismatch repair genes (green).

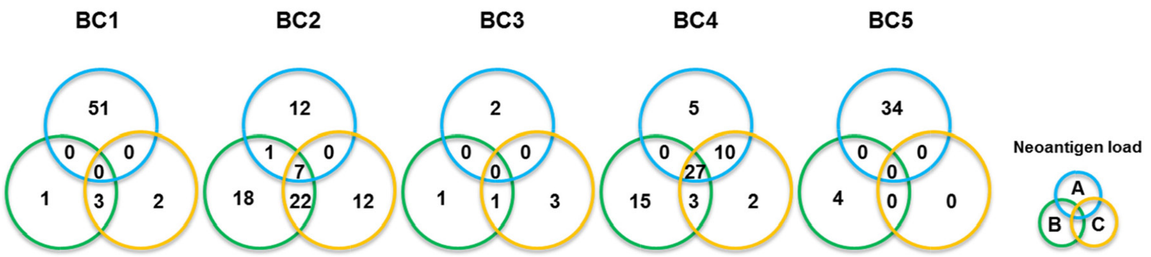

Supplementary Figure 2: Venn diagrams show the distribution of the number of predicted neoantigens in each portion.

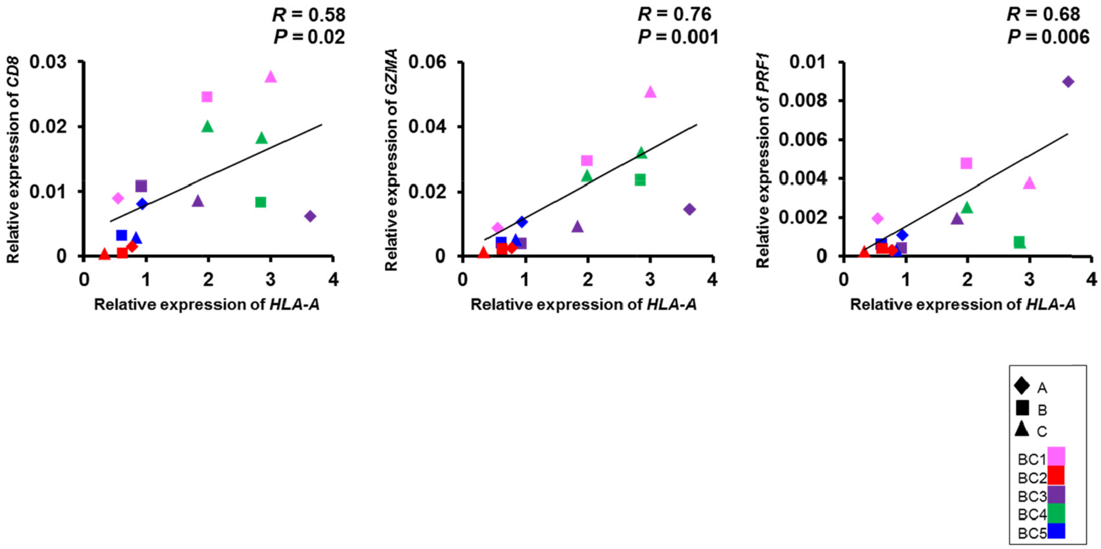

Supplementary Figure 3: Correlation analysis between the expression level of HLA-A and **(A)** CD8, **(B)** GZMA and **(C)** PRF1 in five breast tumors ($N = 15$).

Supplementary Table 1: The list of non-silent mutations and insertions/deletions (indels) in all tumors

See Supplementary File 1

Supplementary Table 2: TCRB sequencing results in all tumors

Patients	Region	Total reads	Obeserved Clonotypes	Unique Clonotypes
BC1	A	157005	131505	15042
BC1	B	7393136	4824822	290001
BC1	C	124807	118812	12589
BC2	A	2500744	479261	18002
BC2	B	1192305	166277	5371
BC2	C	2591518	399926	11680
BC3	A	2349026	1748933	74293
BC3	B	2868165	2401936	153519
BC3	C	4635328	3371886	217928
BC4	A	2253876	1681574	63291
BC4	B	1838145	280571	9873
BC4	C	2328920	1621555	61340
BC5	A	2807328	2160572	164738
BC5	B	1813713	1569877	37196
BC5	C	2375659	1834308	74197
